# Coexistence of “headache attributed to airplane travel” and “mountain descending headache”

**DOI:** 10.1186/1129-2377-14-S1-P169

**Published:** 2013-02-21

**Authors:** F Mainardi, F Maggioni, C Lisotto, G Zanchin

**Affiliations:** 1 Headache Centre, Neurological Division, SS Giovanni e Paolo Hospital, Venice, Italy; 2 Headache Centre, Department of Neurosciences, Padua University, Padua, Italy; 3 Headache Centre, San Vito al Tagliamento Hospital, San Vito al Tagliamento, Italy

## Introduction

Recently, we have published a paper on our study of “Airplane headache” (AH) features in a large series of patients [1]. AH refers to a form of recently described headache disorder, whose attacks are strictly related to airplane travel, mostly to the landing phase.

## Materials and methods

Through a detailed questionnaire we identified, in our population of 85 AH cases, 11 patients suffering from headache attacks also occurring during the rapid descent of mountain by car.

## Results

These patients (5 males, 6 female, mean age 37 years), who have complained of AH attacks during landing in more than 50% of their flights, referred the occasional onset of jabbing, severe, unilateral headaches in the fronto-temporal region when descending a mountain by car. They described the headaches as quite similar, with exactly the same features, as compared with those experienced during landing. No accompanying symptoms were reported. The pain began shortly afterwards the rapid descent by car from a medium altitude of 1,800 metres, the maximum peak of intensity developing in a few minutes. All of them reported the pain subsidence within 20 minutes of the rapid way down. No concomitant airways disturbance was reported during the travel. General and neurological examination, brain MRI, Angio-MRI, and cranial CT-scan for sinuses were normal.

## Conclusions

The coexistence of headache attacks, sharing peculiar features, triggered by these different situations, landing by airplane and descent of high altitude by car, strengthens the hypothesis of a possibly common pathophysiological mechanism, i.e. the change of air pressure, which occurs in both conditions.
